# Supplementary material for: The causal role of circulating inflammatory markers in osteoporosis: a bidirectional Mendelian randomized study
Source: Front Immunol. 2024 Jul 18;15:1412298. doi: 10.3389/fimmu.2024.1412298 (PMC11291241; doi:10.3389/fimmu.2024.1412298)
Supplement: Supplementary file 1 [file DataSheet_1.zip › supplementary material/OSTEOPOROSIS-CIM/GCST90274819.tsv.gz_forest.pdf]

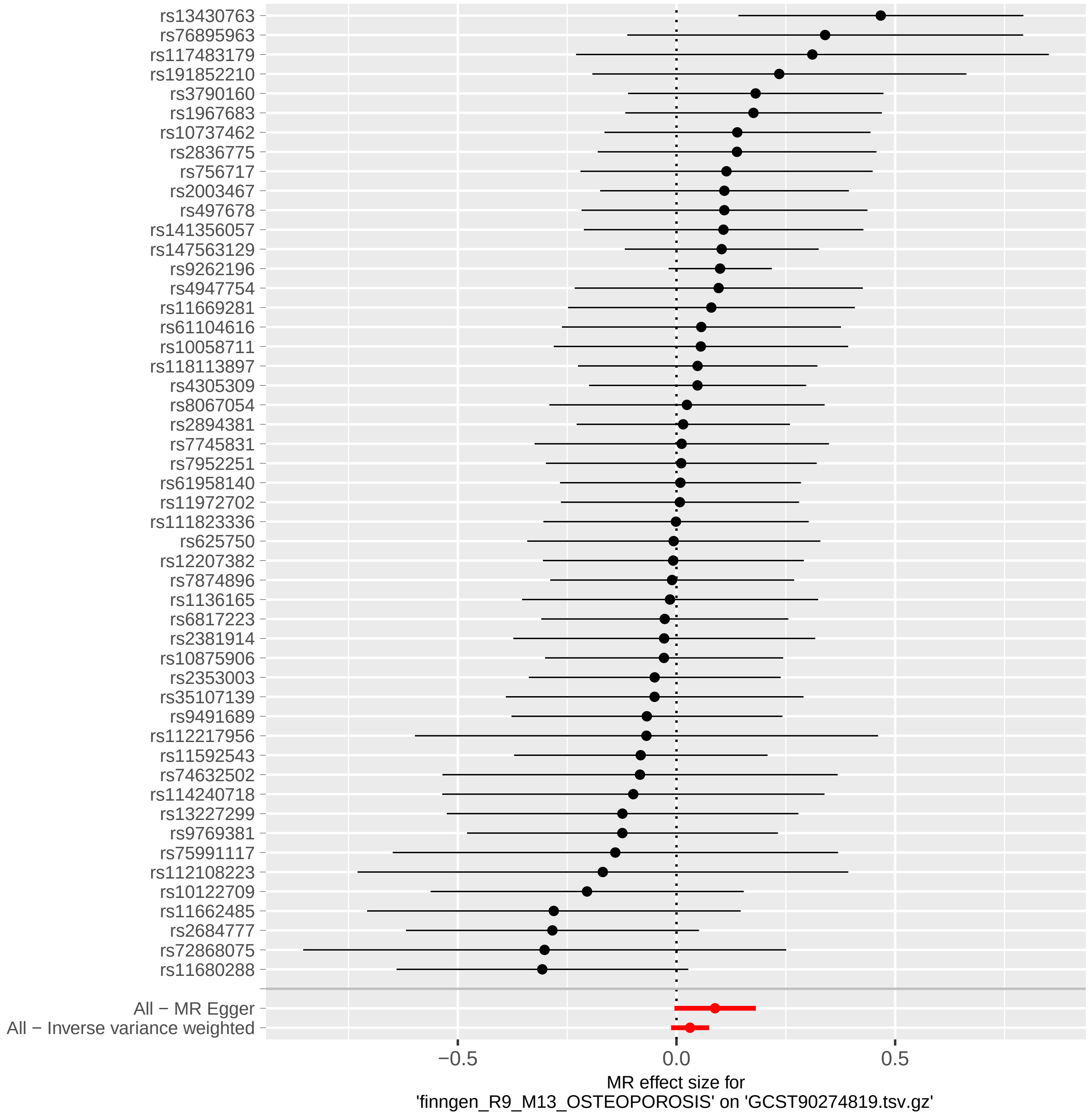
rs13430763
rs76895963
rs117483179
rs191852210
rs3790160
rs1967683
rs10737462
rs2836775
rs756717
rs2003467
rs497678
rs141356057
rs147563129
rs9262196
rs4947754
rs11669281
rs61104616
rs10058711
rs118113897
rs4305309
rs8067054
rs2894381
rs7745831
rs7952251
rs61958140
rs11972702
rs111823336
rs625750
rs12207382
rs7874896
rs1136165
rs6817223
rs2381914
rs10875906
rs2353003
rs35107139
rs9491689
rs112217956
rs11592543
rs74632502
rs114240718
rs13227299
rs9769381
rs75991117
rs112108223
rs10122709
rs11662485
rs2684777
rs72868075
rs11680288
All − MR Egger
All − Inverse variance weighted
−0.5
0.0
0.5
MR effect size for
'finngen_R9_M13_OSTEOPOROSIS' on 'GCST90274819.tsv.gz'
